# Preputial Graft Ureteroplasty for the Treatment of Complex Ureteral Stricture: A New Surgical Technique and Review of Literature

**DOI:** 10.1089/cren.2018.0055

**Published:** 2018-08-01

**Authors:** Bulent Onal, Mehmet Hamza Gultekin, Muhammed Fatih Simsekoglu

**Affiliations:** Department of Urology, University of Istanbul Cerrahpasa School of Medicine, Istanbul, Turkey.

**Keywords:** graft, preputium, stricture, ureter, ureteroplasty

## Abstract

***Objectives:*** To present our first experience and 12-month outcomes of the novel technique of onlay preputial graft ureteroplasty (PGU) for complex ureteral stricture.

***Methods:*** In December 2016, open onlay PGU was made on a male patient who have proximal stricture of the right ureter. The length of upper ureteral stricture was 50 mm. A 60 mm in length and 15 mm in width preputial graft was harvested from the ventral side of the penis and placed in the ureter as a ventral onlay for ureteroplasty. Operative time, intraoperative, and postoperative complications were recorded properly. Follow-up was performed via clinical assessment of symptoms, renal ultrasound, MR urography, and nuclear scan renography.

***Results:*** The new technique was effectively performed without any intraoperative and postoperative complications. Residual hydronephrosis in the right side was found through ultrasonography 3, 6, and 12 months and MR urography 6 months postoperatively. But complaint of the patient's pain passed completely.

***Conclusions:*** Within our knowledge, we present the first experience with onlay PGU for proximal ureteral stricture. Twelve-month results indicate that the new technique appears to be an excellent option for proximal ureteral stricture. Our experience is encouraging, and it will find wider application in the complex ureteral lesion.

## Introduction

In recent years, many urologic diseases have been effectively treated with minimally invasive endourologic procedures. With the increase of endoscopic treatment, iatrogenic ureteral injuries have begun to arise. Ureterorenoscopy-related injuries vary from simple ureteral perforation to avulsion. This most serious and dramatic complications often result with ureteral stenosis. Early diagnosis and treatment of ureteral strictures is important to preserve kidney function. Age of the patient, condition of contralateral kidney, localization, length and etiology of ureteral stenosis, and surgical experience of the surgeon are important factors in determining the treatment option. Considering proximal ureteral strictures, ureteroureterostomy, transureteroureterostomy, ureterocalicostomy, boari flap, psoas “hitch,” intestine interposition, renal autotransplantation, and nephrectomy are some of the treatment methods available.^1^

In this article, we present a preputial graft urethroplasty procedure in a patient who referred to our clinic with ureteral stenosis, who had the previous ureterorenoscopy resulted with ureteral avulsion due to a ureteral stone and had many consecutive surgical procedures regarding to repair ureteral avulsion.

## Presentation of Case

### History and physical examinations

A 37-year-old male patient who had no history of stone disease in his own and family had presented with right flank pain. On CT scan, 5 mm proximal ureteral stones had been detected and ureterorenoscopy had been performed 2 years ago. Perioperatively, stone had been trapped in the basket catheter, and while it had been attempted to be rescued, full-length complete avulsion of ureter had occurred. When the ureterorenoscope was removed from the urethra, the segment had been found on the ureterorenoscope (scabbard avulsion). At the same operation, avulsed ureter had been anastomosed over the J stent in proximal (pyeloureterostomy) and distal (ureteroureterostomy). The patient had been reoperated in postoperative first month due to the extensive leakage of the drain. The distal ureter had been found to be necrotic and this segment had been excised, kidney had been mobilized, and ureteroureterostomy had been reperformed in the same manner. The removal of J stent in the postoperative 4 weeks resulted in a flank pain and hydronephrosis. During the following 3 months, the patient had undergone ureteral balloon dilatation and J stent replacement twice.

### Diagnosis

At 6 months after the first procedure, the patient was admitted to our clinic, and two severe proximal ureteral strictures were detected in a segment of 5 cm (Fig. 2A). The patient was informed about the operation and informed consent was taken. The patient was interned to our clinic, and preputial graft ureteroplasty (PGU) was planned.

### Intervention

Cystoscopy and retrograde pyelography were performed, and two severe strictures in a segment of 5 cm in the proximal ureter were demonstrated.

Open surgery was preferred instead of laparoscopy since our patient has multiple scars due to supraumbilical, infraumbilical, and pelvic Gibson incisions performed in the previous operations. We used a supra- and infraumbilical midline incision.

The ipsilateral colon was moved medially, the ureter was identified, and stricture segment was dissected free. Due to previous distal necrotic ureter excision and kidney mobilization, all urinary tract was adherent to the adjacent strictures. When the ureter was appropriately exposed, the dilatation of proximal ureter was used as a guide and the ureter was incised ventrally for the entire length of the stricture (Fig. 2B). This incision was expanded by scissors to reveal at least 5 mm of healthy ureter proximal and distal to the stenosis. Upper ureteral stricture length was 50 mm. A 4.8F Double-J stent was placed into the ureter.

### Preputial graft harvesting

**FIG. 1.**
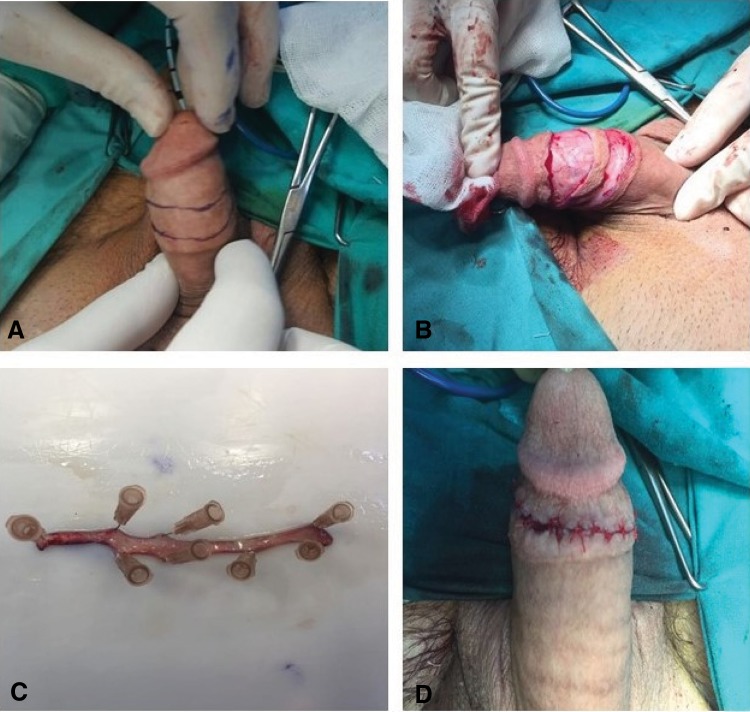
**(A)** Marking of penis incision line. **(B)** Excision of preputial graft. **(C)** Preparing of preputial graft. **(D)** Suturing of incision line.

The site of the harvesting graft on the ventral side of the penis was marked (Fig. 1A). The graft was excised by scissor (Fig. 1B). Submucosal muscle and adipose tissue were excised to create an ultrathin patch graft to be fed through diffusion (Fig. 1C). The incision line was sutured separately with 5/0 poliglactin 910 (Fig. 1D).

### Preputial graft ureteroplasty

**FIG. 2.**
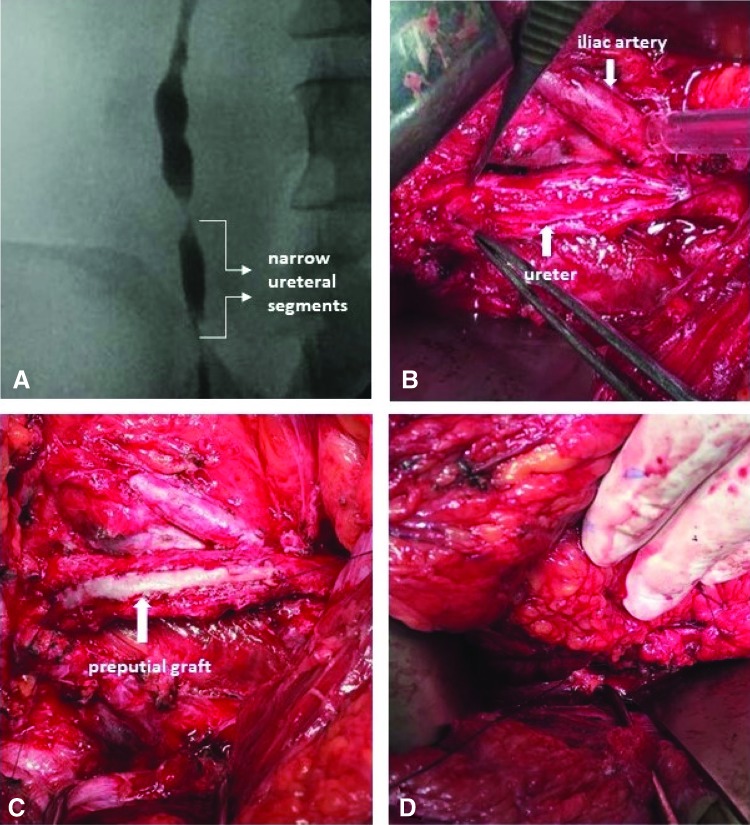
**(A)** Narrow ureteral segments viewed on retrograde pyelography. **(B)** Incision of narrow ureteral segments. **(C)** Anastomose of preputial graft to the ureter as onlay flap. **(D)** The area of repair covering with wrap.

The edges of the graft were anastomosed tension freely and water tightly to the ureter as an onlay flap in running manner, using 4.0 polyglactin sutures (Fig. 2C).

The area of repair was liberally covered with omental wrap, with the intent to augment vascularity (Fig. 2D). A 10 mm drain tube was placed in the retroperitoneum, and abdomen was closed in layers.

### Outcome

**FIG. 3.**
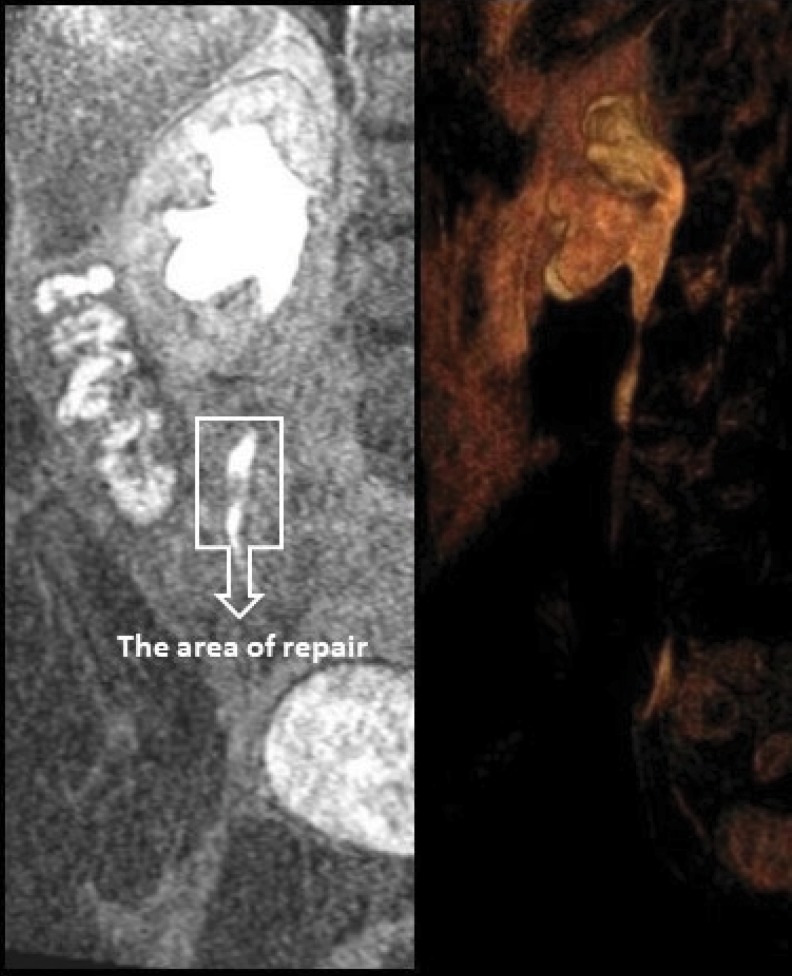
Right ureter images in MR urography at postoperative control.

The postoperative recovery was uneventful, and the drain was removed on the sixth day after surgery. Three months later, the stent was removed, and retrograde pyelography showed patency of the ureter without evidence of obstruction. Residual hydronephrosis and minimal ureteral dilatation in the right side were found through ultrasonography 3, 6, and 9 months and MR urography 6 months postoperatively. Also, renal function and ureteral urine flow was normal in MAG 3 renal scintigraphy and MR urography (Fig. 3). Complaint of the patient's pain passed completely.

## Discussion

Ureteral strictures are challenging situations even for experienced surgeons and often require complex urologic surgeries. Considering proximal ureteral strictures, ureteroureterostomy, transureteroureterostomy, ureterocalicostomy, boari flap, psoas “hitch,” intestine interposition, renal autotransplantation, and nephrectomy are some of the treatment methods available.^1^ For functionally normal kidney, nephrectomy is not easily accepted by the surgeon and the patient. In addition, the ileal ureter interposition may cause many perioperative and postoperative complications, such as intestinal leaks, mucous plugging, stone formation, electrolyte disturbance, recurrent urinary tract infection, and renal dysfunction. Appendiceal interposition has been defined as an alternative to come up from those complications. A possible disadvantage of using the appendiceal interposition is its limited length. Renal autotransplantation was first described in 1963 by J.D. Hardy to a patient with a ureter injury.^2^ Early urinary leakage, rupture in ureterovesical anastomosis, bleeding, and vascular thrombosis are some of the complications. This procedure requires high surgical experience.

Moreover, buccal mucosal grafts (BMG) are used for the treatment of ureteral strictures. In 1999, Naude first described the use of BMG for treatment of long ureteral strictures, in a case series of six patients.^3^ This technique was used with a success in open and robot-assisted laparoscopic procedure.

Barbagli et al. had popularized the effective use of preputial skin in the treatment of bulbourethral strictures^4^ and also preputial graft, which is a well-known tissue for urethral reconstruction. Preputial/distal penile skin is devoid of hair and fat and hence an ideal graft material. Even in circumscribed patients distal penile skin can be harvested. In addition, harvesting and using of preputial graft is easier than buccal mucosal graft. The use of preputial graft for ureteral replacement is minimally invasive when comparing the other options such as bowel interposition and kidney autotransplantation even using the buccal mucosa graft. To our knowledge, PGU for ureteral stricture has not been previously described.

## Conclusion

We herein describe our first experience and 12-month outcomes with open PGU. The new technique appears to be an excellent option for proximal ureteral stricture and allows us to make a tension-free watertight anastomosis by preserving the ureteral vascularity. We believe that PGU can be used safely for the repair of ureteral strictures and our experience is encouraging the clinicians for the treatment of complex ureteral lesion. A larger set of studies and longer follow-up are needed to evaluate and optimize this procedure.

## Abbreviations Used

BMG: buccal mucosal grafts
CT: computed tomography
PGU: preputial graft ureteroplasty
